# Increased excitability and synaptic transmission of GABAergic neurons derived from a patient with alternating hemiplegia of childhood

**DOI:** 10.1186/s13041-026-01338-7

**Published:** 2026-08-11

**Authors:** Min-Gyun Kim, Hien Bao Dieu Thai, Woojoong Kim, Sol Choi, Heeju Kim, Kyoung-Doo Hwang, Jangsup Moon, Yong-Seok Lee, Byung Chan Lim

**Affiliations:** 1 Department of Physiology, Seoul National University College of Medicine, 103 Daehak-ro, Jongno-gu, Seoul, 03080 Republic of Korea; 2 Department of Biomedical Sciences, Seoul National University College of Medicine, Seoul, Republic of Korea; 3 Department of Pediatrics, Pediatric Clinical Neuroscience Center, Seoul National University Children’s Hospital, 101 Daehak-ro, Jongno-gu, Seoul, Seoul, 03080 Republic of Korea; 4 Department of Neurology, Seoul National University Hospital, Seoul, Republic of Korea; 5 Medical Research Center, Neuroscience Research Institute, Seoul National University, Seoul, Republic of Korea

**Keywords:** *ATP1A3*, GABAergic neuron, Alternating hemiplegia of childhood, Induced pluripotent stem cell

## Abstract

Mutations in *ATP1A3*, which encodes the α3 subunit of the Na⁺/K⁺-ATPase, are associated with various neurological disorders, including alternating hemiplegia of childhood (AHC). Although several studies have used mouse models to investigate *ATP1A3* function, their translational relevance remains limited due to species-specific differences in neurodevelopment and neural circuitry. Additionally, previous patient-derived induced pluripotent stem cell (iPSC) models were limited by their focus on excitatory neurons. Given the prominent expression of the α3 isoform in GABAergic neurons, we investigated GABAergic neuron cell type-specific dysfunction using AHC-patient-derived iPSC. This iPSC was generated from the urinary cells of a control subject and a patient with AHC harboring a heterozygous *ATP1A3*^E815K/+^ mutation and subsequently differentiated into GABAergic neurons. AHC-patient-derived GABAergic neurons exhibited significantly reduced ouabain-sensitive pump currents, indicating a loss-of-function effect of the mutation. Pump deficiency is accompanied by intrinsic hyperexcitability and increased spontaneous inhibitory synaptic transmission. These findings provide direct evidence of GABAergic neuronal dysfunction in AHC and highlight the importance of GABAergic pathology in *ATP1A3*-related disorders.

**Supplementary Information:**

The online version contains supplementary material available at https://doi.org/10.1186/s13041-026-01338-7.

## Introduction

*ATP1A3* mutations lead to a heterogeneous group of neurological disorders collectively referred to as *ATP1A3*-related disorders. Following the discovery of *ATP1A3* mutations as the causative genetic basis of rapid-onset dystonia-Parkinsonism in 2004, the associated clinical spectrum and genetic landscape have broadened substantially [1–3]. Despite insight into the genetic etiology, the precise mechanisms that translate *ATP1A3* dysfunction into the complex paroxysmal phenotypes observed in patients remain incompletely understood.

Emerging evidence from both human transcriptional analyses and animal models suggests that GABAergic neurons play an essential role in the pathophysiology of *ATP1A3*-related disorders [4–6]. Within the mammalian brain, the α3 isoform is reported to be prominently expressed in GABAergic neurons of the basal ganglia, hippocampus and somatosensory cortex [7, 8]. Furthermore, in vivo studies utilizing *ATP1A3* mutant mouse models have revealed the loss of GABAergic neurons, leading to hippocampal hyperexcitability, supporting the hypothesis that *ATP1A3*-related disorders are fundamentally diseases of GABAergic dysfunction [5]. However, despite accumulating evidence, human-specific data regarding GABAergic dysfunction remain unclear. To date, patient-derived induced pluripotent stem cell (iPSC) models of *ATP1A3*-related disorders have largely been restricted to the study of excitatory neurons, leaving a critical gap in our understanding of how *ATP1A3* mutations alter inhibitory signaling and network circuitry [9, 10].

To bridge this gap, it is essential to investigate the functional consequences of *ATP1A3* mutations in GABAergic neurons. Therefore, we used a directed stepwise differentiation protocol to generate a highly enriched population of developing GABAergic neurons from iPSC derived from a patient with alternating hemiplegia in childhood harboring the c.2443G > A (p.E815K) mutation [11]. The E815K variant is the second most common *ATP1A3* mutation and is clinically notable for causing the most severe alternating hemiplegia of childhood (AHC) phenotype, characterized by early neonatal onset, severe intellectual and motor disabilities, and drug-resistant epilepsy [2]. By characterizing the electrophysiological properties of these AHC-patient-derived *ATP1A3*-mutant developing GABAergic neurons, this study provides evidence of GABAergic neuronal dysfunction associated with an *ATP1A3* mutation.

## Methods

### Human subjects and ethics

A urine sample (~ 20 mL) was collected with written informed consent from a patient with genetically confirmed AHC harboring a heterozygous mutation (*ATP1A3*^E815K/+^, hereafter referred to as “mutant line”) and an unrelated healthy individual (*ATP1A3*^+/+^, hereafter referred to as “control line”). This study was reviewed and approved by the Institutional Review Board of the Seoul National University Hospital (IRB 1905-116-1035 and 2210-115-1372).

### Patient-derived iPSC generation from urinary cells (UCs)

UCs were isolated from patient urine samples and reprogrammed into iPSC as previously described by Thai et al., with modifications to the protocol reported by Zhou et al. [12, 13]. In brief, pelleted cells were seeded onto gelatin-coated 12-well plates in primary urinary culture medium, then gradually transitioned to a 1:1 mixture of Renal Epithelial Medium and Mesenchymal Cell Medium until 80–90% confluency. UCs were subsequently reprogrammed into iPSC by transfection with episomal vectors (Epi5 Kit; Invitrogen) containing plasmids encoding OCT3/4, SOX2, KLF4, L-MYC, and LIN28, followed by electroporation. The reprogrammed cells were seeded onto Matrigel-coated plates, sequentially cultured in proliferation medium supplemented with a Rho-associated coiled-coil kinase (ROCK) inhibitor, followed by N2B27 medium, and transferred to mTeSR Plus medium (STEMCELL Technologies) on day 15. The iPSC-like colonies became visible between days 21 and 30 and were then transferred to Matrigel-coated 6-well plates for expansion. To generate representative iPSC lines for each donor, at least three independent polyclonal iPSC pools were established, each of which was generated by manually picking and pooling 3–5 morphologically ideal colonies from a primary reprogrammed plate. The resulting cultures were expanded and characterized for iPSC pluripotency; genomic integrity was assessed using chromosomal microarray prior to cryopreservation. Validated iPSC were preserved as independent iPSC parental lines.

### iPSC characterization and validation

 Pluripotency was confirmed via immunocytochemistry for the markers OCT3/4, NANOG, SSEA-4, and TRA-1-60 following 4 days of growth on Matrigel-coated coverslips (1:400). The antibodies used in this study are listed in Supplementary Table 1.

Functional pluripotency was assessed using an embryoid body (EB) differentiation assay. To form EB, a 90% confluent iPSC monolayer was dissociated with 2 mg/mL collagenase IV for 30–45 min at 37 °C, and resuspended in Embryoid body-KnockOut (EB-KO) medium (KnockOut DMEM (Gibco), KnockOut serum replacement (KOSR; Gibco), 1% NEAA, 1% GlutaMAX, 1% A/A, and 55 µM β-mercaptoethanol).

Cells were pelleted and transferred to a 6-well low-attachment plate (Corning) for EB formation, and the medium was changed every 48 h. On day 8, EBs were plated onto a 0.1% gelatin-coated 24-well plate in EB-fetal calf serum (FCS) medium (similar to EB-KO, but the KOSR was replaced with FCS). On day 16, differentiation into the three germ layers was validated via staining for smooth muscle actin (SMA; mesoderm), alpha-fetoprotein (AFP; endoderm), and β3-tubulin III (ectoderm).

The genomic integrity of iPSC was assessed using chromosomal microarray (CMA) analysis as previously described [13].

### Stepwise differentiation of iPSC to ventral forebrain GABAergic neurons

To differentiate iPSC into ventral forebrain GABAergic neurons, the 70-day protocol of Liu et al. was refined with key optimizations for consistency and reproducibility [10]. Neural induction (Days 0–6) was initiated via dual SMAD inhibition during EBs formation at defined cell densities in 96-well plates. Ventral patterning in neural progenitors (Days 7–16) was followed by the substitution of recombinant Sonic hedgehog (SHH) protein with the small-molecule agonist purmophamine. To guarantee morphological uniformity, the neurospheres were cultured (Days 17–25) in 384-well plates before plating the intact cells on a poly-l-ornithine (PLO)/laminin-coated coverslip for terminal differentiation (Days 26–70) into mature GABAergic neurons. These modifications, including defined seeding densities in 96- and 384-well formats and small molecule-mediated patterning, enabled the generation of highly consistent GABAergic neuronal cultures. The detailed differentiation protocol was as follows:

#### Days 0–6: neural induction

iPSC (80% confluent) was dissociated with gentle cell dissociation reagent (STEMCELL Technologies) and re-seeded at 9000 cells/well in ultra-low-attachment 96-well plates (Corning) to form EBs in EBs formation medium (DMEM/F12 medium (Gibco), 20% KOSR, 1% Glutamax, 1% NEAA, 50 µM β-mercaptoethanol) supplemented with dual SMAD inhibitor (10 µM SB431542, 10 µM DMH-1). ROCK inhibitor (10 µM) was then added to the medium for the subsequent 24 h. The medium was changed daily until day 3. From days 4 to 6, half of the medium was replaced with a neural induction medium (NIM; DMEM/F12 medium containing 1% N_2_ supplement, 1% NEAA and 5 µg/mL heparin).

#### Days 7–16: medial ganglionic eminence (MGE) progenitor’s generation

EBs were collected and plated onto matrigel-coated plates (1:100) in NIM supplemented with 5% FBS for 6 h, then switched to serum-free NIM. Fresh NIM medium was replaced every two days. From day 11 onward, 1.5 µM Purmorphamine (Pur; REPROCELL) was added to induce ventral forebrain MGE-like progenitors via SHH signaling activation. Purmorphamine, a small-molecule SHH pathway agonist, was used instead of recombinant SHH protein for cost efficiency.

#### Days 17–25: neurospheres formation

Neural rosettes were isolated by incubation in Rosette Selection Reagent (STEMCELL Technologies) for 1 h, followed by washing in NIM for collection, and centrifugation at 1200 rpm for 3 min. The resulting pellet was resuspended in NIM and seeded at a density of 2000 cells/well into low-attachment 384-well black plates (Corning) in NIM medium supplemented with 1% N_2_ and 2% B27 for additional culture for 10 days.

#### Days 26–70: terminal differentiation of cells into forebrain ventral GABAergic neurons

Intact neurospheres were plated directly onto PLO/laminin-coated coverslips without any further dissociation. The cells were matured in neuron differentiation medium (NDM; Neurobasal plus medium (Gibco) containing 1% Glutamax, 1% NEAA, 1% N_2_, 2% B27, 1 µM cAMP (Sigma-Aldrich), 10 ng/mL BDNF (Peprotech), 10 ng/mL GDNF (Peprotech), and 10 ng/mL hIGF (Peprotech). Half of the medium was replaced every three days, and neurons were further differentiated and matured for an additional six weeks.

### Immunostaining and imaging

 Cells were fixed in 4% paraformaldehyde (Biosesang) for 30 min at room temperature, permeabilized with 0.3% Triton X-100 (Sigma-Aldrich) in PBS for 15 min, and blocked with 5% goat serum (Gibco) for 1 h. Cells were incubated with primary antibodies overnight at 4 ℃, followed by incubation with secondary antibodies for 2 h, and nuclei counterstaining with DAPI for 10 min. Fluorescent signals were captured using a Leica confocal microscope and analyzed using LAS X Office software. The antibodies used in this study are listed in Supplementary Table 1.

### Electrophysiology

Whole-cell patch-clamp recordings were performed at 70–77 days in vitro as previously described [14]. Recordings were conducted at room temperature under continuous perfusion with artificial cerebrospinal fluid (ACSF; in mM: 125 NaCl, 2.5 KCl, 1 MgCl₂, 2 CaCl₂, 1.25 NaH₂PO₄, 26 NaHCO₃, 10 glucose) and equilibrated with 95% O₂/5% CO₂ for at least 15 min prior to experiments.

For intrinsic excitability measurements, glass pipettes with resistances of 4–5 MΩ were filled with potassium-based intracellular solution (in mM: 135 K-gluconate, 5 KCl, 2 NaCl, 10 HEPES, 0.1 EGTA, 5 Mg-ATP, 0.4 Na₂-GTP, 10 Tris-phosphocreatine; pH 7.20 adjusted with KOH) were used. For synaptic current measurements, a cesium-based intracellular solution was employed (in mM: 140 Cs-methanesulfonate, 7 NaCl, 10 HEPES, 0.2 EGTA, 4 Mg-ATP, 0.3 Na₂-GTP). Signals were acquired using an EPC-9 patch-clamp amplifier (HEKA Elektronik) using PatchMaster software, sampled at 20 kHz, and low-pass filtered at 2 kHz; series resistance was maintained below 20 MΩ. Synaptic events were analyzed using custom LabVIEW scripts (https://github.com/parkgilbong/Minhee_Analysis_Pack).

For ouabain-sensitive current measurements, pipettes were filled with intracellular solution (in mM: 95 Na-methanesulfonate, 20 TEA-Cl, 2 BaCl₂, 5 EGTA, 4 Mg-ATP, 5 Tris-phosphocreatine, 10 HEPES, 5 glucose; pH 7.35 with NaOH, ~ 280 mOsm/kg adjusted with sucrose). Cells were superfused with a K⁺-free extracellular solution (in mM: 140 NaCl, 1 MgCl₂, 2 BaCl₂, 2 CsOH, 2 NiCl₂, 0.2 CdCl₂, 0.33 NaH₂PO₄, 5 HEPES, 5 glucose, 20 NMDG-Cl; pH 7.35 with HCl). The total pump current was elicited by switching to a solution in which 20 mM NMDG-Cl was substituted with 20 mM KCl; ouabain-sensitive current was isolated by repeating activation in the presence of 10 µM ouabain. The cells were voltage-clamped to + 40 mV throughout.

For the intrinsic excitability measurements, neurons were obtained from three independent differentiation batches. Neurons were obtained from two independent differentiation batches for synaptic and ouabain-sensitive current measurements. Within each batch, data were collected from neurons differentiated on 20 individual coverslips (representing 20 distinct cell cultures).

### Statistical analysis

Data are presented as mean ± SEM. Statistical analyses were performed using GraphPad Prism version 9. Two-group comparisons were performed using unpaired two-tailed Student’s t-tests. Repeated-measures two-way analysis of variance (ANOVA) followed by Bonferroni’s multiple comparison test was used for current or cohort analyses. The units of analysis were individual cells obtained from independent differentiation batches, as indicated in the figure legends.

## Results

### Generation of patient-derived iPSC from UCs

Patient-derived UCs were successfully generated from urine samples obtained from a healthy donor and a patient with AHC harboring the *ATP1A3*^*E815K/+*^ mutation within approximately 10 days (Supplementary Fig. 1A). Subsequently, UCs were programmed into iPSC using a virus-free and non-integrating episomal vector system that was successfully employed and validated in a previous study [13]. Aggregation of reprogrammed cells was observed from day 10 post-electroporation, followed by vigorous proliferation and emergence of well-defined iPSC-like colonies with a distinct flat, rounded appearance on day 25 (Supplementary Fig. 1B). The iPSC colonies were manually isolated and expanded to establish stable iPSC for further analysis. The pluripotency of these stable iPSC was further verified at both the phenotypic and functional levels. Immunocytochemical analysis indicated strong expression of the pluripotency markers OCT3/4, NANOG, TRA-1-60, and SSEA-4 (Supplementary Fig. 2A), wherein the direct differentiation of spontaneous embryoid bodies revealed the capability of iPSC to differentiate into derivatives of all three germ layers: the ectoderm, mesoderm, and endoderm (Supplementary Fig. 2B). The genomic integrity of iPSC was assessed using CMA, confirming their suitability for subsequent differentiation experiments.

### Directed differentiation of iPSC into ventral forebrain GABAergic neurons

To investigate the specific role of GABAergic neurons in AHC pathogenesis, we differentiated *ATP1A3*^*E815K/+*^ and control iPSC into an enriched population of ventral forebrain GABAergic neurons over 70-day period (Supplementary Fig. 3A). Briefly, early neural induction was initiated by culturing aggregated iPSC as suspension EBs through dual SMAD inhibition—targeting transforming growth factor-β (TGFβ) and bone morphogenetic protein (BMP) signaling. This approach suppresses mesodermal and endodermal lineages while promoting the formation of primitive neuroepithelial cells during the first five days of differentiation [15]. Subsequently, EB was plated on a Matrigel-coated plate, and ventral patterning was induced via activation of SHH signaling using purmophamine. Radially organized neuroepithelial rosette structures subsequently mature into 3D neurospheres, which serve as a pool of MGE-like neural progenitor cells (NPCs). Upon placement on the PLO/laminin-coated coverslips, the neurospheres exhibited vigorous migration and profound morphological changes (Supplementary Fig. 3B). At 35 days in vitro (DIV35), the cells exhibited a temporal progression of neuronal migration and neurite outgrowth from the NPCs. By DIV56, the neurons displayed significant increases in axonal length and soma size, forming dense, interconnected neuritic networks and complex neuronal clusters, indicative of ongoing neuronal differentiation.

**Fig. 1:**
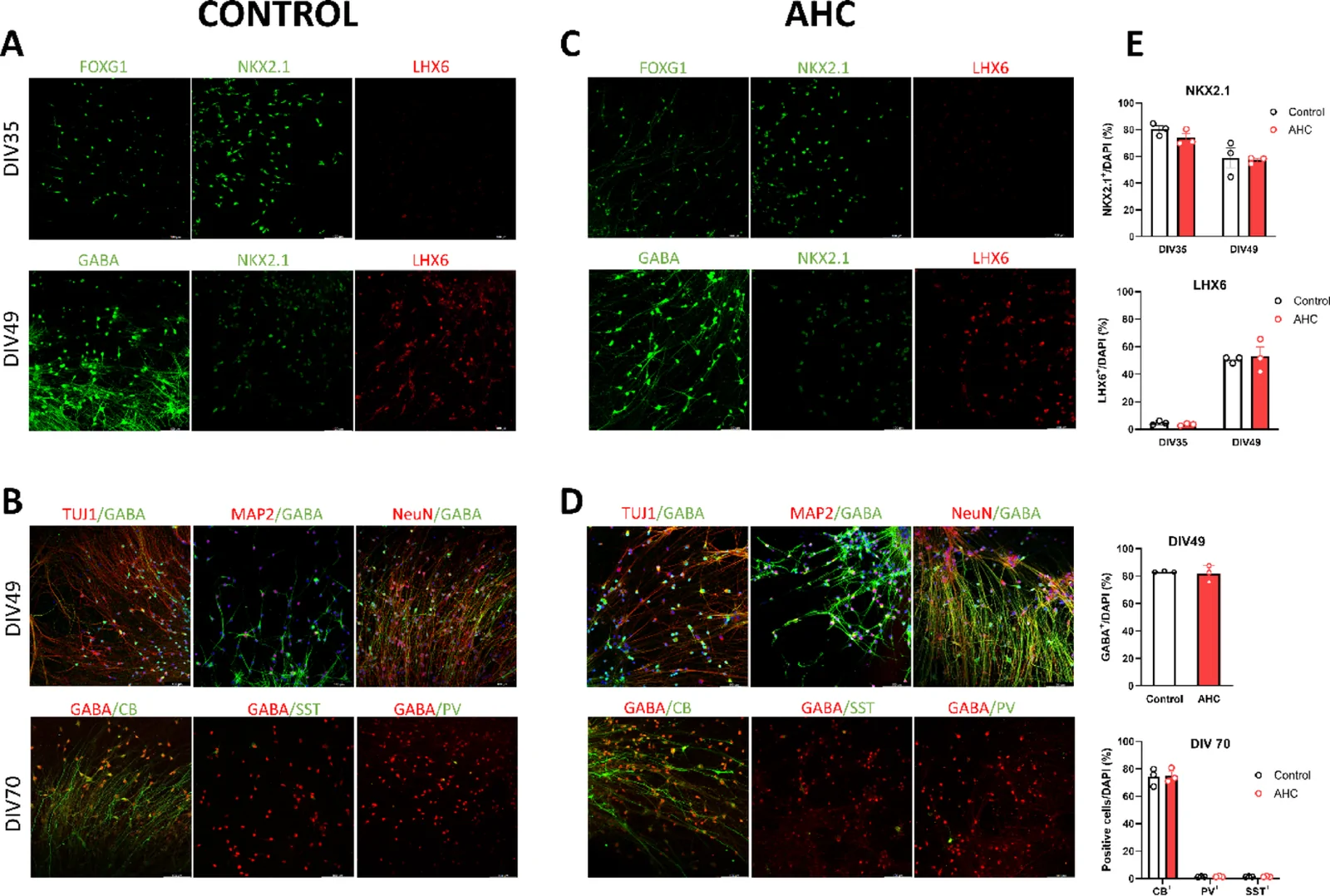
Specification of ventral forebrain GABAergic identity in induced pluripotent stem cell (iPSC)-derived neurospheres. **A**, **C** Immunofluorescence staining for regional and neuronal markers in neurospheres and differentiated neurons. Staining includes FOXG1 (telencephalic forebrain), NKX2.1 (ventral forebrain/early medial ganglionic eminence (MGE) marker), LHX6 (MGE late marker), and GABA (GABAergic neurotransmitter). **B**, **D** Co-staining for neuronal maturation and interneuronal subtype markers. TUJ1/GABA, MAP2/GABA, NeuN/GABA, calbindin (CB)/GABA, somatostatin (SST)/GABA, and parvalbumin (PV)/GABA were present in both control and mutant lines. **E** Quantification of neural markers in control and alternating hemiplegia of childhood (AHC) mutant cells. Data are presented as mean ± SEM of 3 independent biological replicates

To assess the molecular maturation and morphological changes, immunocytochemical analyses were conducted at various stages of differentiation. At the early stage (DIV35), both control and mutant cultures robustly expressed the forebrain marker FOXG1 and the early MGE progenitor marker NKX2.1 (control: 80.3 ± 2.6%, AHC: 73.7 ± 3.3%; Fig. 1A, C, E). At this stage, LHX6 expression was minimal (control: 4.6 ± 0.8%, AHC: 3.45 ± 0.4%; Fig. 1A, C, E), which is consistent with an early MGE progenitor state. By DIV49, NKX2.1 expression was markedly reduced (control: 59.1 ± 7.4%, AHC: 57.2 ± 1.3%; Fig. 1A, C, E) and LHX6 was strongly upregulated (control: 50.6 ± 1.3%, AHC: 52.9 ± 6.8%; Fig. 1A, C, E), indicating progression to a late MGE-derived GABAergic neuronal identity. The ventral forebrain MGE-derived GABAergic cell identity was further validated by robust GABA expression in 83.1 ± 0.2% and 79 ± 3.6% of cells in control and AHC cultures, respectively (Fig. 1A, C, 1E). This was accompanied by the expansion of neuronal networks and upregulation of pan-neuronal maturation markers, including TUJ1, MAP2, and NeuN, indicating progressive neuronal differentiation and maturation (Fig. 1B and D). GABAergic neuron subtype analysis revealed an early to-intermediate maturation pattern. Calbindin (CB), a marker of immature interneurons, is predominantly expressed during DIV49 (control: 74.1 ± 3.7%, AHC: 74.9 ± 2.9%). However, terminally differentiated MGE-derived markers, the expression of somatostatin (SST, control: 1.3 ± 0.1%, AHC: 1.4 ± 0.2%) and parvalbumin (PV, control: 1.2 ± 0.1%, AHC: 1.5 ± 0.2%), remained at minimal levels throughout DIV70 (Fig. 1B, D, E). Extended maintenance of the neurospheres up to DIV85 did not result in any significant increase in SST or PV expression. Instead, we observed a deterioration in culture integrity, characterized by weakened network organization and reduced cell density (data not shown). Therefore, we decided to use the developing GABAergic neurons at DIV70-77 for subsequent experiments.

### AHC-patient-derived GABAergic neurons exhibit reduced ouabain-sensitive current

**Fig. 2:**
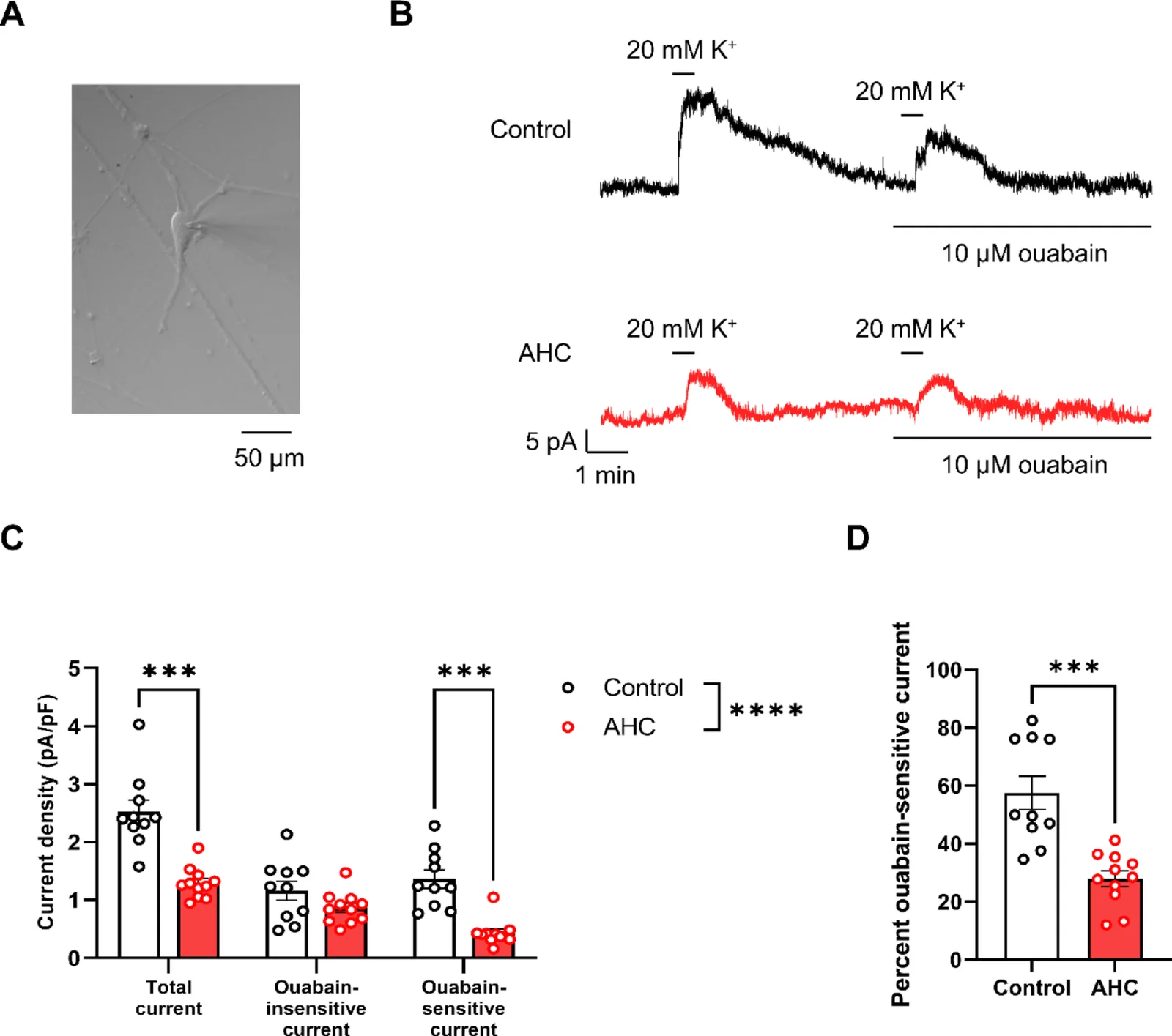
AHC-patient-derived GABAergic neurons exhibit reduced ouabain-sensitive currents. **A** A representative bright field image of an AHC-patient-derived GABAergic neuron contacted by a patch pipette for whole-cell patch clamp recording. **B** Whole-cell currents were activated by 20 mM extracellular potassium in the absence or presence of 10 µM ouabain from either control (black) or AHC-patient-derived (red) GABAergic neurons. Holding potential was + 40 mV. **C** Summary of current density for total, ouabain-insensitive and ouabain-sensitive currents measured from control (white) or AHC-patient-derived (red) GABAergic neurons. Ouabain sensitive current was obtained by subtracting the ouabain-insensitive current from the total current. (control, *n* = 10 cells from 2 batches; AHC, *n* = 11 cells from 2 batches; repeated measures of two-way ANOVA (study cohort x current component): study cohort effect F1,19 = 33.44, ****p < 0.0001, with Bonferroni’s multiple comparison test, total current; ***p < 0.001, ouabain-sensitive current; ***p < 0.001). **D** Summary column plots for percent ouabain-sensitive currents measured from control (white) or AHC-patient-derived (red) GABAergic neurons (control, *n* = 10 cells from 2 batches; AHC, *n* = 11 cells from 2 batches; unpaired t test, ***p < 0.001). Data are expressed as means ± SEM

To investigate the electrophysiological effect of the *ATP1A3* mutation on Na^+^/K^+^-ATPase function in developing GABAergic neurons, we quantified the ouabain-sensitive current elicited by extracellular potassium in control or AHC-patient-derived GABAergic neurons using whole-cell patch-clamp recording (Fig. 2A). Under saturated intracellular sodium, both total currents and ouabain-sensitive currents were significantly attenuated in response to 20 mM extracellular potassium in AHC-patient-derived GABAergic neurons compared with control neurons (Fig. 2B–D). Notably, repeated application of 20 mM extracellular potassium induced comparable whole-cell currents, indicating that the effects of ouabain were not due to inactivation of Na^+^/K^+^- ATPase activity (Supplementary Fig. 4).

### Altered intrinsic excitability and membrane properties in AHC-patient-derived GABAergic neurons

**Fig. 3:**
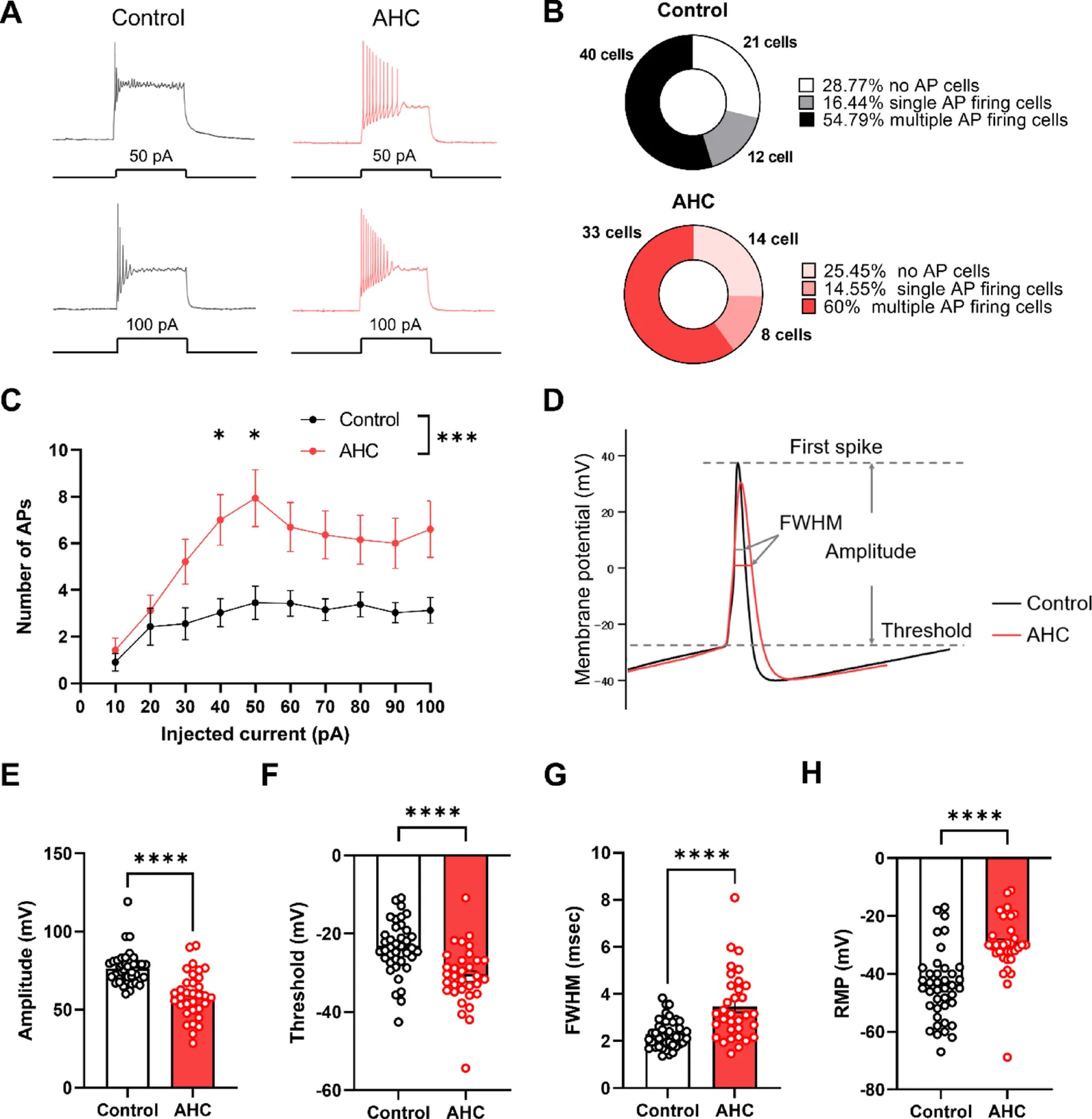
AHC-patient-derived GABAergic neurons show significant changes in resting membrane potential and action potential properties. **A** Intracellular current injection elicited distinct patterns of action potential (AP) firing in control (black, *n* = 73 cells from 3 batches) and AHC-patient-derived (red, *n* = 55 cells from 3 batches) GABAergic neurons. Somatic current steps ranged from 10 to 100 pA (10 pA increments) with a 1-second stimulus duration. **B** Recorded cells were classified by AP firing frequency. Neurons displaying repetitive AP generation were selected for subsequent electrophysiological characterization. **C** AP firing frequency revealed increased excitability in AHC-patient-derived GABAergic neurons compared with controls (Control, *n* = 40 cells from 3 batches; AHC, *n* = 33 cells from 3 batches; repeated measures of two-way ANOVA (study cohort x injected current): study cohort effect F1,71 = 13.00, ***p < 0.001, with Bonferroni’s multiple comparison test, 40 pA, 50 pA; *p < 0.05; others were n.s.). **D** The first spike elicited in representative neurons was analyzed to calculate the electrophysiological parameters of the action potential: threshold, peak amplitude, and full-width at half-maximum (FWHM). **E**–**H** Passive and active properties of multiple AP firing neurons were analyzed. AHC-patient-derived GABAergic neurons showed spike broadening and elevated resting membrane potential (RMP) (Control, *n* = 40 cells from 3 batches; AHC, *n* = 33 cells from 3 batches; unpaired t test, ****p < 0.0001)

To examine the intrinsic properties of the AHC-patient-derived developing GABAergic neurons, we performed current-clamp recordings. Intracellular current injection (10–100 pA, 10 pA increments, 1 s duration) elicited action potential (AP) firing in both control and AHC-patient-derived GABAergic neurons (Fig. 3A). Human iPSC-derived GABAergic neurons undergo progressive maturation during in vitro differentiation, and AP firing emerges in a subset of neurons as they acquire more mature neuronal properties [16, 17]. Based on this distinction, neurons were classified as having no, single, or multiple AP firing cells. Subsequent analyses focused on multiple AP firing, which represents a relatively mature subpopulation within the cultures, rather than a fully mature interneuron population (Fig. 3B). AHC-patient-derived GABAergic neurons exhibited an increased firing frequency in response to incremental current injections compared to control-derived GABAergic neurons (Fig. 3C). AHC-patient-derived GABAergic neurons exhibited decreased AP peak amplitude and threshold, while the full width at half maximum (FWHM) of the AP increased (Fig. 3D–G). Additionally, the augmented resting membrane potential (RMP) demonstrated that AHC-patient-derived GABAergic neurons were depolarized (Fig. 3H). These changes in passive and active properties suggest altered intrinsic excitability in AHC-patient-derived developing GABAergic neurons carrying the *ATP1A3* mutation.

### Inhibitory synaptic transmission is increased in AHC-patient-derived GABAergic neurons

**Fig. 4:**
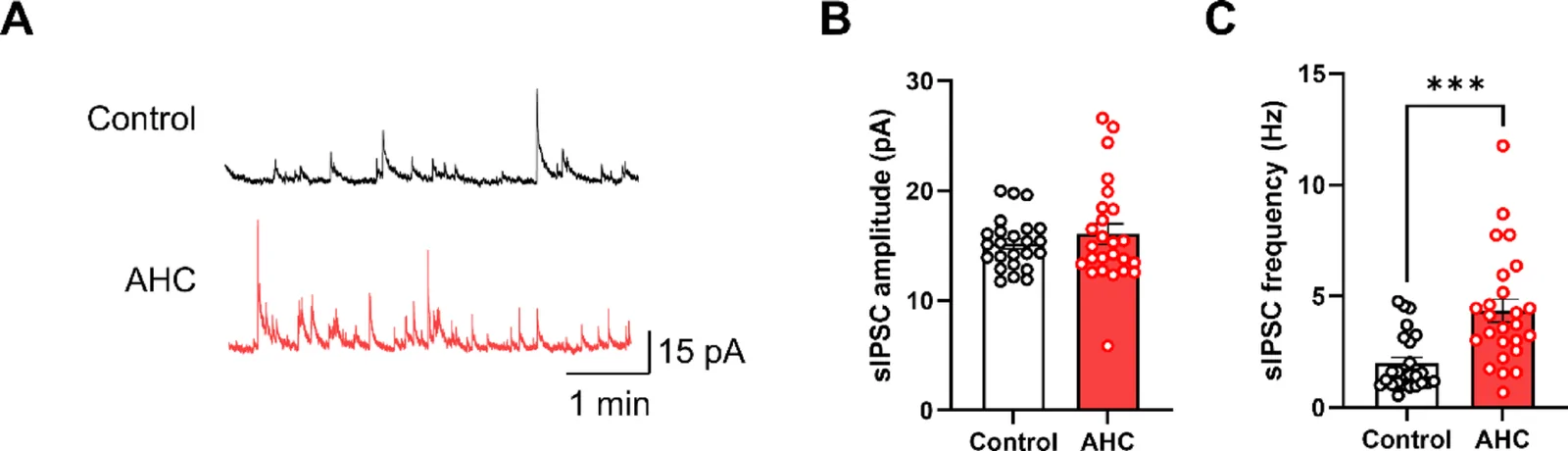
Increased spontaneous inhibitory postsynaptic current (sIPSC) frequency in AHC-patient-derived GABAergic neurons. **A** Representative traces of inhibitory postsynaptic currents are shown. **B** sIPSC amplitudes were comparable between control and AHC-patient-derived GABAergic neurons. **C** The frequency of sIPSC (Control, *n* = 24 cells from 2 batches; AHC, *n* = 25 cells from 2 batches; unpaired t test, ***p < 0.001). Data are expressed as means ± SEM

Because most of these neurons were GABAergic, we examined spontaneous inhibitory postsynaptic currents (sIPSCs) in AHC-patient-derived GABAergic neurons (Fig. 4A). AHC-patient-derived GABAergic neurons exhibited a significant increase in sIPSC frequency compared with control neurons, whereas sIPSC amplitudes remained comparable between the two groups (Fig. 4B–C). These data indicate that inhibitory synaptic transmission was augmented in AHC-patient-derived GABAergic neurons.

## Discussion

In this study, we successfully differentiated iPSC collected from an AHC patient (*ATP1A3*^*E815K/+*^) and a healthy individual into enriched populations of GABAergic neurons and detailed their electrophysiological profiles. By utilizing a protocol to mimic the in vivo development of the MGE, our model provides information on the cell type-specific vulnerability of AHC-patient-derived GABAergic neurons. AHC-patient-derived GABAergic neurons exhibit impaired Na^+^/K^+^-ATPase function, which increases intrinsic excitability and action potential firing frequency. Furthermore, increased frequency of sIPSCs is accompanied by intrinsic hyperexcitability. Taken together, our results indicate that *ATP1A3* mutations may lead to a pathological increase in the GABAergic output.

Although the cultures showed robust expression of GABA and neuronal markers, the relatively low expression of mature interneuron subtype markers, including SST and PV, indicated that these cells were more appropriately interpreted as developing GABAergic neurons than as fully mature interneurons. Therefore, the electrophysiological changes observed in AHC-patient-derived cultures should be considered alterations in developing GABAergic neurons rather than direct evidence of mature interneuron dysfunction.

The intrinsic properties of AHC-patient-derived GABAergic neurons are in sharp contrast to previous iPSC studies that predominantly utilized excitatory neurons. Consistent with our observations, a previous iPSC study using *ATP1A3*^G947R/+^ excitatory neurons showed Na^+^/K^+^-ATPase pump dysfunction and depolarized RMP [9]. However, in their excitatory model, chronic depolarization resulted in reduced excitability and lower firing rates. This was explained as a depolarization block, in which voltage-gated sodium channels enter a state of steady-state inactivation owing to elevated RMP. Similarly, *ATP1A3*^E815k/+^ iPSC study using excitatory neurons demonstrated reduced baseline activity and hyperactivity following heat stress [10]. Our data indicate that GABAergic neurons do not experience this depolarization block at baseline. Instead, the pump defect is converted into increased baseline excitability and sustained high-frequency firing, highlighting a critical cell type-specific divergence in pathophysiology.

The discrepancy between our findings and those of studies using excitatory neuron models likely stems from the unique electrophysiological properties of developing GABAergic neurons. Unlike pyramidal neurons, which express Nav1.2 and are prone to slow inactivation during depolarization, developing GABAergic neurons expressing Nav1.1 exhibit resistance to both slow inactivation and use-dependent inactivation [18–20]. Consequently, moderate resting membrane depolarization driven by Na^+^/K^+^-ATPase dysfunction recruits these available channels to drive repetitive firing rather than inducing the sodium channel inactivation observed in pyramidal neurons. In addition, early-stage CB^+^ GABAergic neurons have high input resistance and membrane time constants, resulting in increased sensitivity to external current input [21, 22]. Taken together, these results indicate that early developing GABAergic neurons are likely to exhibit cell type-specific responses to Na^+^/K^+^-ATPase pump dysfunction.

Our observations are consistent with those of previous animal models, indicating that *ATP1A3* dysfunction enhances GABAergic transmission. Enhanced inhibitory synaptic neurotransmission localized to the presynaptic axonal terminus was observed in the cerebellar cortex of younger *ATP1A3* knockout mice (postnatal days 12–15). The authors proposed a presynaptic augmentation mechanism, in which Na^+^/K^+^-ATPase pump failure leads to terminal calcium accumulation, resulting in excessive GABA release [23].

Conversely, other studies have reported decreased GABAergic transmission in *ATP1A3* mutant mice at later developmental stages. For example, one study found that *ATP1A3*⁺^/^ᴰ⁸⁰¹ᴺ knock‑in mice (postnatal day 21 ~ 30) exhibited reduced GABAergic inhibition, as indicated by attenuated spontaneous IPSCs and evoked IPSCs without any changes in evoked excitatory postsynaptic currents (EPSCs). Consistent with these findings, PV^+^ interneuron density decreased in the hippocampal CA1 region of ATP1A3^+/D801N^ knock-in mice [5]. Similarly, the lowered seizure threshold in *ATP1A3*^+/D801Y^ knock‑in mice (Postnatal day 70 ~ 140) was rescued by clonazepam, indicating that decreased GABAergic transmission underlies the heightened network excitability observed in these mutant mice [4, 24]. Findings from early-stage rodent models and iPSC-based in vitro models appear to contradict those from later-stage rodent models, indicating that *ATP1A3* dysfunction may exert opposing effects on GABAergic signaling, depending on the developmental context. Whether this apparent discrepancy reflects any genuine developmental transition in how *ATP1A3* dysfunction manifests, or merely the differences between species and model systems, currently remains an important open question.

This study provides the first direct evidence of intrinsic hyperactivity in human *ATP1A3*-mutant GABAergic neurons during early developmental stages. Overall, we revealed that developing GABAergic neurons do not succumb to a depolarization block, as in previous excitatory neuron iPSC models, but instead exhibit pathological hyperexcitability. Because *ATP1A3* is expressed in the early embryonic stage, our study suggests that the pathological impact of *ATP1A3* mutations likely affects the early maturation of GABAergic neurons.

One limitation of this study is the lack of donor-level replication, as the GABAergic neuron model was generated from a single patient with AHC carrying the *ATP1A3*^E815K/+^ mutation and a single unrelated healthy control. Although multiple electrophysiological abnormalities consistently support a disease-relevant phenotype, donor-specific genetic backgrounds, clonal variations, epigenetic effects, and differences in differentiation potential cannot be excluded. Therefore, our findings should be considered proof-of-concept evidence of GABAergic neuronal dysfunction associated with *ATP1A3*^E815K/+^ rather than definitive evidence of a generalized AHC phenotype.

Having established intrinsic dysfunction in the early development of human GABAergic neurons, future studies must address how this continuous hyperexcitability affects neuronal subtype specification and cortical-subcortical circuit wiring. Since GABAergic neuron maturation depends heavily on local network interactions and excitatory drives, single-lineage purified cultures are insufficient for studying later developmental stages [25]. Future studies should employ heterogeneous mixed cell-type models that faithfully recapitulate the late prenatal and early postnatal human brain environments.

## Supplementary Information

Below is the link to the electronic supplementary material.

Supplementary Material 1.

Supplementary Material 2.

## Data Availability

All data supporting the findings of this study are presented within the article. No additional data is generated.

## Abbreviations

AFP: Alpha-fetoprotein
AHC: Alternating hemiplegia of childhood
AP: Action potential
BMP: Bone morphogenetic protein
CB: Calbindin
CMA: Chromosomal microarray
DIV: Days in vitro
EB: Embryoid body
EPSCs: Excitatory postsynaptic currents
FCS: Fetal calf serum
FWHM: Full-width at half-maximum
iPSC: Induced pluripotent stem cell
MGE: Medial ganglionic eminence
NPCs: Neural progenitor cells
PLO: Poly-l-ornithine
PV: Parvalbumin
RMP: Resting membrane potential
SHH: Sonic hedgehog
sIPSCs: Spontaneous inhibitory postsynaptic currents
SMA: Smooth muscle actin
SST: Somatostatin
TGFβ: Transforming growth factor-β
UCs: Urinary cells
