# Supplementary material for: Increased excitability and synaptic transmission of GABAergic neurons derived from a patient with alternating hemiplegia of childhood
Source: Mol Brain. 2026 Aug 11;19:80. doi: 10.1186/s13041-026-01338-7 (PMC13741543; doi:10.1186/s13041-026-01338-7)
Supplement: Supplementary file 1 [file 13041_2026_1338_MOESM1_ESM.pdf]

Supplementary Table 1. The antibodies used in the experiment

Antibody	Source	Catalog No.	Dilution
**OCT3/4**	Abcam	ab18976	1:200
**NANOG**	Thermo Fisher	MA5-15469	1:200
**TRA-1-60**	Thermo Fisher	MA1-023	1:200
**SSEA-4**	Thermo Fisher	41-4000	1:200
**FOXG1**	Abcam	ab18259	1:1000
**NKX2.1**	Sigma-Aldrich	MAB5460	1:1000
**LHX6**	Bioss	bs-7869R	1:1000
**GABA**	Sigma-Aldrich	A2052	1:1000
**TUJ1 (βIII-tubulin)**	Sigma-Aldrich	T8660	1:1000
**MAP2**	Abcam	ab32454	1:1000
**NeuN**	Sigma-Aldrich	MAB377	1:1000
**Calbindin (CB)**	Abcam	ab108404	1:200
**Somatostatin (SST)**	Abcam	ab313450	1:500
**Parvalbumin (PV)**	Abcam	ab11427	1:1000
