# Supplementary material for: Increased excitability and synaptic transmission of GABAergic neurons derived from a patient with alternating hemiplegia of childhood
Source: Mol Brain. 2026 Aug 11;19:80. doi: 10.1186/s13041-026-01338-7 (PMC13741543; doi:10.1186/s13041-026-01338-7)
Supplement: Supplementary file 2 [file 13041_2026_1338_MOESM2_ESM.pdf]

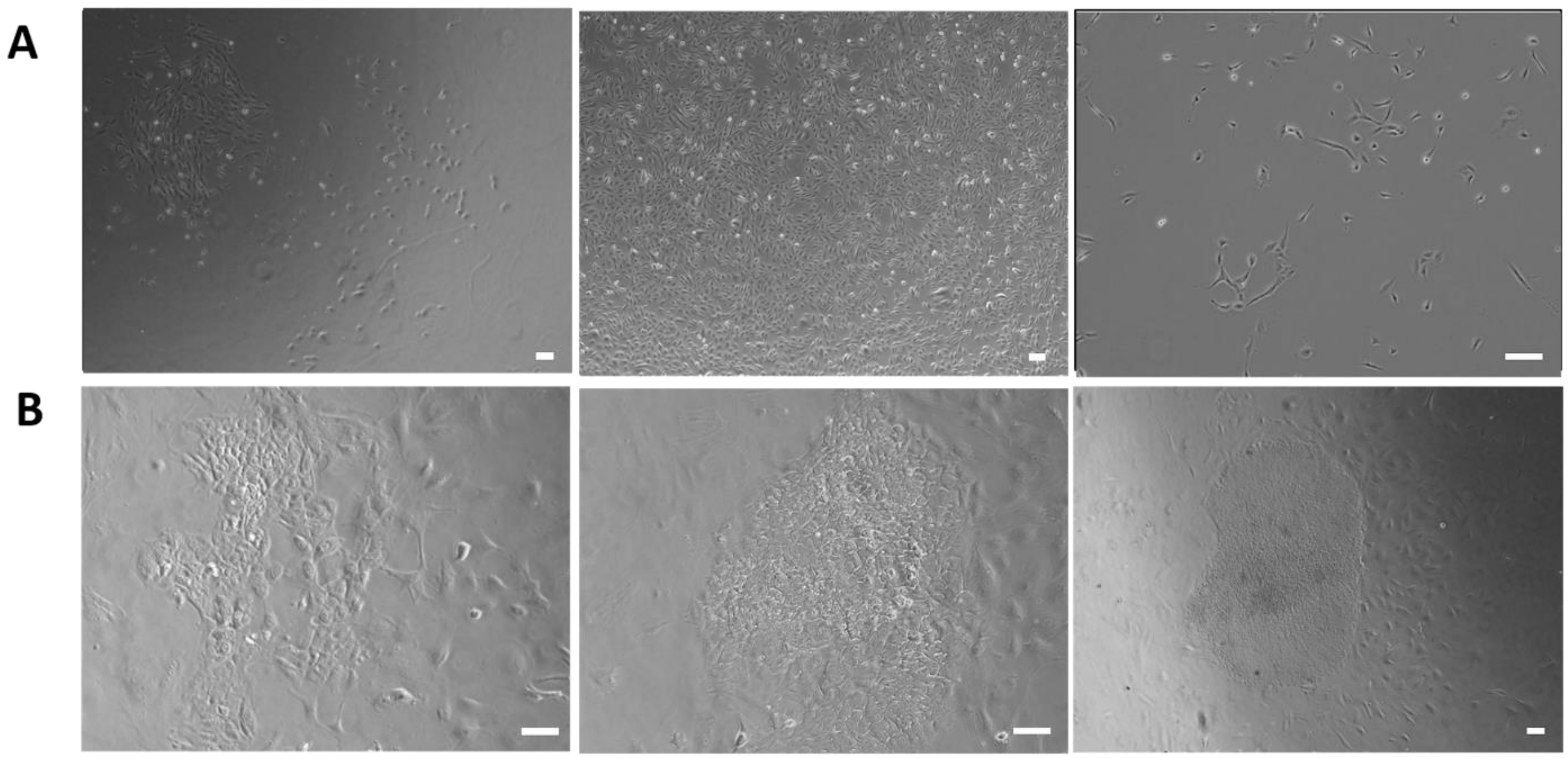

Supplementary Figure 1. **Generation of induced pluripotent stem cell (iPSC) from urine.** **A**. Morphology during urine cell isolation and reprogramming. Left panel: Urine cells at day 4. Middle panel: Urine cells at day 15. Right panel: Urine cells reprogrammed to iPSC. **B**. iPSC colony progression in feeder-free conditions. Left panel: iPSC in early stage formation by reprogrammed urine cells. Middle panel: iPSC aggregation. Right panel: iPSC ready to pick and passage. Scale bar, 100 µm.

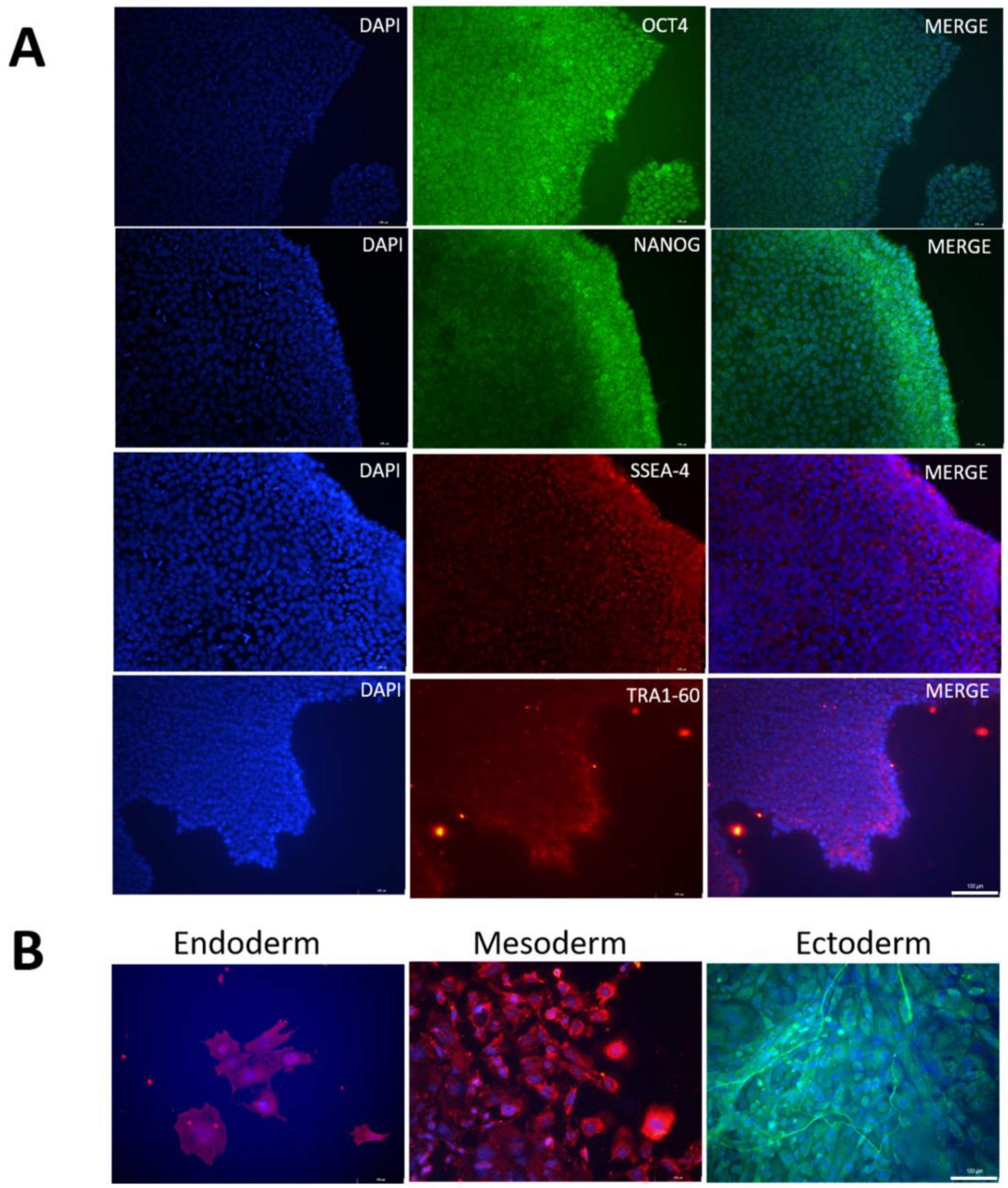

Supplementary Figure 2. Characterization of iPSC generated from urine.

Immunofluorescence staining of iPSC and embryoid body differentiation. **A.** Positive staining for pluripotency markers OCT4, SOX2, NANOG, SSEA-4, and TRA-1-60, with DAPI nuclear counterstaining. **B.** Representative differentiation images showing markers of the three germ layers: Endoderm (AFP), Mesoderm (SMA), and Ectoderm (β-Tubulin III). Scale bar, 100 μm.

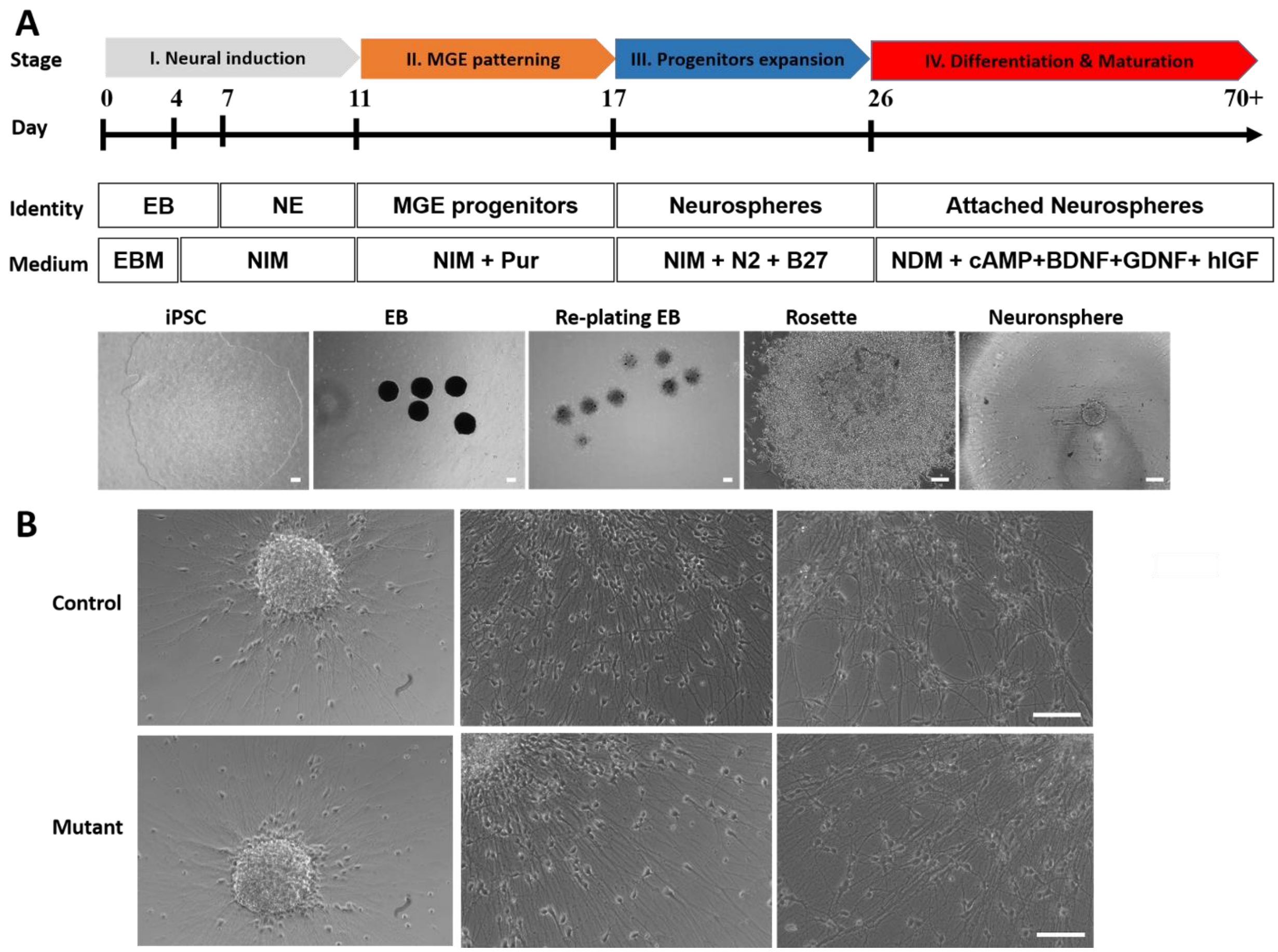

Supplementary Figure 3. Stepwise differentiation of iPSC into ventral forebrain GABAergic neurons.

A. Schematic timeline and representative images illustrating the sequential stages of GABAergic neuron generation from human iPSC (hiPSC). **B.** Brightfield images of neurospheres plated onto poly-L-ornithine (PLO)/laminin-coated coverslips from healthy control and mutant lines. Images are shown from left to right at DIV27, DIV35, and DIV56 (DIV, days in vitro). Scale bar, 100 μm.

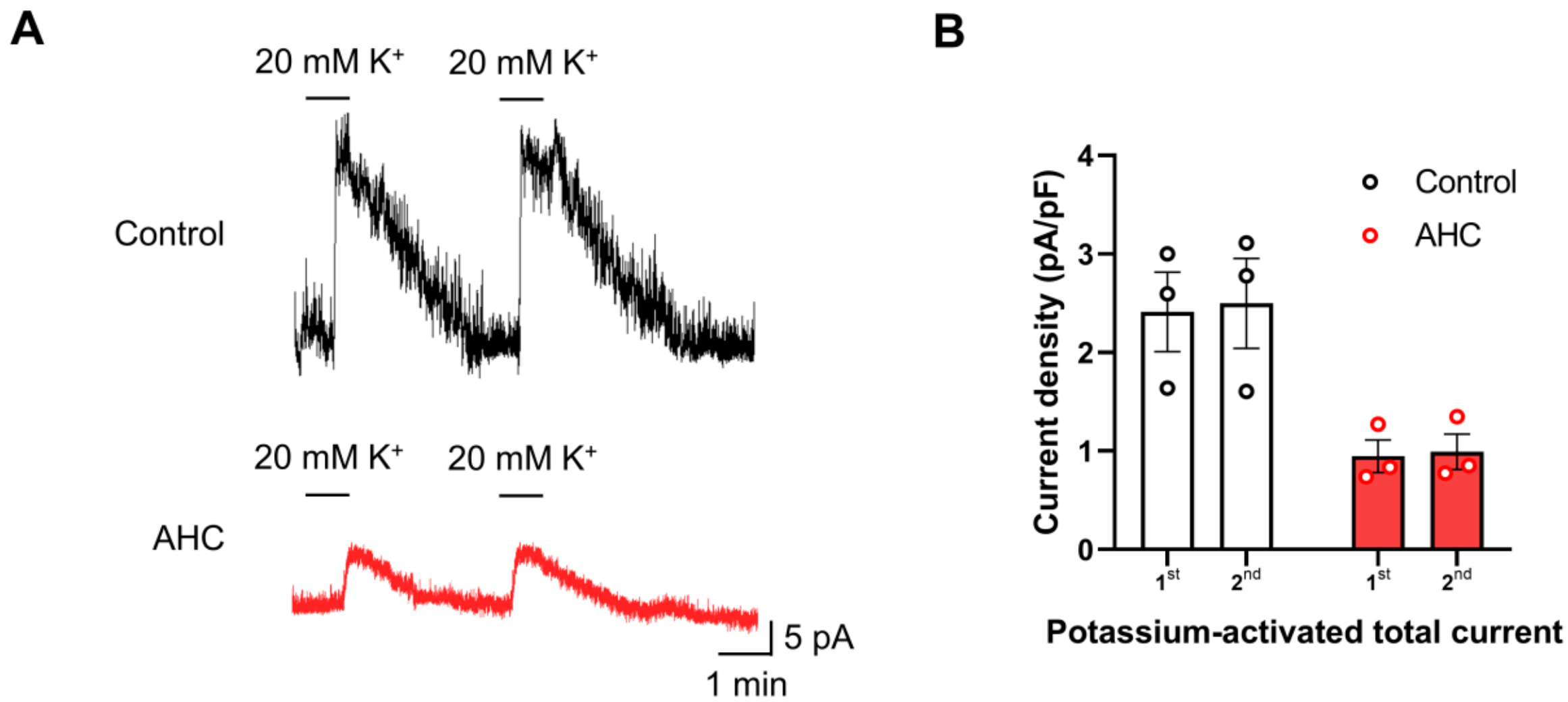

Supplementary Figure 4. Two consecutive applications of 20 mM potassium induce comparable whole-cell currents.

A. Whole-cell currents were recorded from control (black) and alternating hemiplegia of childhood (AHC)-patient-derived GABAergic neurons in response to two consecutive applications of 20 mM extracellular K^+. **B.** Consecutive potassium treatment elicited comparable whole-cell currents in both control and AHC-patient-derived GABAergic neurons (Control, n=3 cells from a single batch; AHC, n=3 cells from a single batch; repeated measures of two-way ANOVA (study cohort x current component): current component effect $F_{1,4} = 3.87$, $p = 0.1205$). Data are expressed as means ± SEM.
